# Behavioral Inhibition and Fear: The Moderating Role of Emotional Competence and Gender in Preadolescents

**DOI:** 10.3390/children12020179

**Published:** 2025-01-31

**Authors:** Andrea Baroncelli, Stefania Righi, Carolina Facci, Enrica Ciucci

**Affiliations:** 1 Department of Philosophy, Social Sciences and Education, University of Perugia, 06123 Perugia, Italy; andrea.baroncelli@unipg.it; 2 Department of Neuroscience, Psychology, Drug Research and Child’s Health, University of Florence, 50139 Florence, Italy; stefania.righi@unifi.it; 3 Department of Education, Languages, Interculture, Literatures and Psychology, University of Florence, 50135 Florence, Italy; carolina.facci@unifi.it

**Keywords:** BIS, attentional facilitation index, emotion awareness, emotion perception accuracy, fear

## Abstract

Background: The behavioral inhibition system (BIS) is a key motivational system that shapes human emotions and behaviors; specifically, the BIS regulates avoidance behaviors, and it is linked to negative emotions such as fear and anxiety. Previous studies have demonstrated a link between high BIS scores and attentional bias to threat in children, but literature is inconsistent. This may be due to differences in the individual awareness of emotions or in the accuracy of effectively detecting emotions. Moreover, the past literature has also found gender differences in BIS scores, which may suggest differential processes in boys and girls. Methods: The present study aims to investigate whether BIS scores were associated with an attentional facilitation index of fear in a sample of preadolescents (*n* = 264; 52.27% girls; M age = 12.98 years; SD = 0.89 years), considering the potential moderating role of (a) the awareness of others’ emotions as assessed by a self-report questionnaire, (b) emotion perception accuracy of fear as assessed by a laboratory task of emotion recognition, and (c) gender. Results: Our results showed that, only in males, higher scores of the BIS were associated with a lower attentional facilitation index of fear in the conditions of low levels of emotional competence (i.e., low levels of self-reported awareness of other emotions or low levels of accuracy recognition of fearful faces). Conclusions: Results were discussed in light of both theories of emotional development and practical clinical implications, with special attention to the emerged gender difference.

## 1. Introduction

Within the field of research investigating individual differences in the processing of emotional stimuli (and, in particular, fear), the aim of the present study is to explore in-depth the association between youths’ level of behavioral inhibition and an attentional facilitation index of fear, considering the potential moderating role of two variables related to emotional competence (i.e., a self-reported awareness of others’ emotions and a measure of accuracy in recognizing fear arising from a laboratory task), as well as gender. Gray’s Reinforcement Sensitivity Theory [1,2,3] posits that individuals differ in the sensitivity of fundamental brain systems that respond to rewards and punishments. Specifically, the theory identifies two core neurobehavioral systems—the behavioral inhibition system (BIS) and the behavioral activation system (BAS)—as keys in motivating behavior and influencing emotional responses [4,5,6]. The BIS primarily responds to cues of punishment, non-reward, or goal conflict [7,8,9], triggering negative emotions such as fear, anxiety, frustration, and sadness; this system drives behavioral inhibition, passive avoidance, heightened arousal, and increased vigilance (e.g., attention and risk assessment) [10,11]. In contrast, the BAS reacts to positive cues like rewards or the absence of punishment, promoting approach-oriented and active behaviors [12]; the activation of this system is associated with the experience of positive emotions and the implementation of reward-seeking behaviors [13]. Conceptually, the BIS operates as an attentional system, scanning for potential threats and regulating responses to negative stimuli [14]. The BAS, on the other hand, functions as a motivational system, responding to rewarding or appetitive signals [15]. In personality studies, the BIS is linked with trait anxiety, while the BAS, initially related to impulsivity, has shown a stronger correlation with extraversion [5,15,16].

Beyond their roles in everyday emotional and personality processes, the extreme activation of the BIS or BAS is linked to risks for various psychological disorders. The BIS over-activation, for instance, is associated with a higher likelihood of experiencing anxiety disorders, depression, or psychosomatic conditions [16,17,18,19]. More specifically, youths with stable and extreme behavioral inhibition are more likely to develop anxiety disorders [20,21,22], including social anxiety [23]. Indeed, the pattern of anxious behaviors, social withdrawal, and negative affect can predict anxiety disorders in adulthood [24]. However, not all children with high behavioral inhibition display inhibition at later ages, and only ~40% go on to develop anxiety problems [23]. As a consequence, intervention efforts need to identify the factors that moderate the stability of behavioral inhibition, as well as the association between early behavioral inhibition and subsequent anxiety. One such factor—which we paid central attention to in the present study—may be attentional bias toward threats.

Attentional bias refers to the propensity to promptly and preferentially attend to threatening stimuli compared to neutral or positive stimuli [25,26,27]. The facilitated detection of stimuli that signify fear or threats (i.e., attentional facilitation) represents an adaptive biological response which may prepare the individual to quickly respond to threatening circumstances; however, when attentional facilitation becomes an attentional bias, it is not adaptive and may compromise the attentional performance because the individuals tend to be hypervigilant even when threats are irrelevant to current goals [28]. Extant research found that an increased detection of threat-related stimuli, such as human faces showing fear, has been specifically linked to heightened anxiety [29,30,31,32], even if the magnitude of this effect and its generalizability to all samples is still under debate and in need of further evidence [25,33,34].

Typically, attentional facilitation (and attentional bias as its high extreme) of a specific emotion has been assessed by the dot-probe task, which is a modified version of the Posner paradigm [35]. The dot-probe task consists of presenting a pair of faces (of the same identity) with different emotional expressions for a constant time period, followed by the display of a visual probe in one of the two faces locations. Usually, one of the faces has a neutral expression and the other has a positive or negative emotional expression. Participants are asked to identify the location of the probe. The underlying assumption of the dot-probe task is that the presence of an emotional face captures the participant’s attention compared to a neutral face, and this can either facilitate or hinder the detection of the dot. Hence, the dot-probe task allows for measuring how much the detection of the dot is facilitated when it is displayed in the same position as the emotional face, and how much it is hindered when the dot is displayed in the opposite position to the emotional face. By manipulating the time interval between the onset of the faces and the appearance of the probe, this task also allows the assessment of both earlier and later attention [36]. To summarize, the increased detection of threat-related stimuli (i.e., a high extreme of attentional facilitation) could play a significant role within the mechanism that connects behavioral inhibition and the development of anxiety disorders in youths. In this regard, there is evidence indicating that adolescents identified as high in behavioral inhibition during childhood showed a stronger attentional bias toward threats compared to their non-inhibited peers [37]. Moreover, children high in behavioral inhibition continued to display inhibited behaviors throughout childhood and adolescence only if they also exhibited a threat attentional bias, therefore sustaining the hypothesis that attentional bias moderates the relationship between behavioral inhibition and anxiety-related outcomes; nevertheless, some other studies failed to find a significant relationship between behavioral inhibition and attentional bias to threats [38,39,40], thus advocating for further research to investigate the association between the BIS and threat attentional bias, including its potential moderators.

### Present Study

Aside from variations in task designs, the above-reported inconsistencies in the association between behavioral inhibition and attentional bias to threats could be due to individual differences in awareness of others’ emotions or in the accuracy of effectively detecting them. In this vein, one previous study [41] found that higher trait emotional intelligence is positively related with BAS scores and positive affect, whereas it is negatively associated with BIS scores and negative affect (anxiety, worry, and rumination). Another study confirmed that greater activation of inhibitory behaviors, associated with goal conflict, was related to more negative affect in subjects with lower emotional intelligence [42]. In general, the awareness of other people’s emotions can have positive effects on the modulation of BIS activation; for example, high emotional intelligence can enhance the motivation to find a job based on the BIS function [43] and can act as a protective factor in sexual victimization, humiliation, and detachment when BIS activation is low [44]. In other words, there are arguments to hypothesize that the BIS can impact differently on (mal)adaptive emotional processes based on different levels of co-occurring emotional competence. Despite the relevance of this hypothesis, to the best of our knowledge, only the very few studies mentioned here [41,42,43,44] have explored the relationship between emotional intelligence and BIS.

Another important point that needs to be addressed is related to the difference in BIS/BAS activation in females and males. In this regard, past studies focused on BIS/BAS activity and associated emotional disorders revealed that females tended to show higher BIS activity and a greater prevalence of affective disorders, including anxiety, depression, and dysthymia [45,46,47]; conversely, males exhibited higher BAS activity and displayed more BAS-related behaviors, such as substance abuse, impulsivity (e.g., ADHD), compulsive behaviors (e.g., pathological gambling), and aggression [48,49,50]. Some studies evidenced that these differences in scores from the BIS/BAS scales can be related to neuroanatomical sex-based differences in activation [51,52]. Nonetheless, considering the complexity of gender as a psychological construct, one possibility is that in addition to differences in BIS/BAS activation, gender stereotypes may also play a main role in mediating emotional responses [53]. Taking into account this consideration, in the present study, we compared the BIS/BAS differences between females and males by considering the construct of gender differences rather than the mere biological sex differences.

Hence, the present study aimed to investigate whether BIS scores were associated with an attentional facilitation index of fear (which, at extreme positive levels, can represent an attentional bias to threat) in a sample of preadolescents, considering the potential moderating role of (a) the awareness of others’ emotions as assessed by a self-report questionnaire, (b) emotion perception accuracy of fear as assessed by a laboratory task of emotion recognition, and (c) gender. This kind of investigation could help in identifying underlying factors associated with both continuity and discontinuity in the expression of behavioral inhibition across development [54,55]. In doing so, since the BIS is a sort of an attentional system scanning for potential threats [14], we hypothesized that higher levels of the BIS were related to a higher attentional facilitation index of fear. Moreover, we hypothesized that lower levels of awareness of others’ emotions or lower levels of the emotion perception accuracy of fear could account for the positive association between the BIS and the attentional facilitation index of fear (i.e., when behavioral inhibition pairs with low emotional competence, the role of the BIS in the attentional facilitation of fear is maximized). Importantly, the joint presence of two emotional competence measures obtained through two different techniques (i.e., a self-report questionnaire and a laboratory task) allowed for the simultaneous testing of both the trait of emotional competence (i.e., awareness of others’ emotions) and the ability of emotional competence (i.e., emotion perception accuracy of fear). Finally, the potential moderating role of gender was tested in an exploratory manner: even if previous research highlighted gender differences in BIS/BAS activity [45,46,47,48,49,50,51,52,53] that suggested the opportunity to test gender roles in the present analyses, there is no argument to advance specific hypotheses.

## 2. Materials and Methods

### 2.1. Participants and Procedure

The present study was conducted using a convenience sample of middle school students coming from Central Italy. Two public schools were first contacted to take part in a research project on emotional development and socioemotional adjustment among middle school students; then, their internal school boards approved their participation and all study procedures. To realize data collection among students, written parental consent was requested for over 350 students; no economic incentives were given.

Data collection was conducted during school hours by trained assistants: students who obtained written parental consent were invited to individually complete a paper booklet of questionnaires during a group session; the emotion recognition tasks were administered to one student at a time in a room that the school had specifically made available for this purpose. Each participant was provided with a code number to control for confidentiality, as well as to maintain the association between the paper booklet of questionnaire and the emotion recognition tasks. Prior to data coding, the following exclusion criteria were applied: unavailability declared by the student to participate in the research; inability to understand written text, even if supported (i.e., presence of an intellectual disability or unfamiliarity with the Italian language); partial completion of measures; and multiple and repeated school failures that placed a student outside of the typical age range (i.e., middle school in Italy is attended by students aged between 11 and 14 years). Consequently, the final sample of the present study consisted of 264 students (52.27% girls; M age = 12.98, SD = 0.89; 92.42% of them were of Italian cultural background; the distribution across the three school grades was grade 6, *n* = 40 (15.15%); grade 7, *n* = 112 (42.42%); and grade 8, *n* = 112 (42.42%)).

### 2.2. Measures

#### 2.2.1. Behavioral Inhibition System

The dispositional BIS was measured using the BIS scale from the BIS/BAS Scales [4], in the Italian version by Leone and colleagues [56]. It is a self-report scale consisting of 7 items that reflect concern over the possibility of a bad occurrence (“I worry about making mistakes”) or responses to such events when they do occur (“Criticism or scolding hurts me quite a bit”). All items were proposed on a 5-point Likert-type scale, with 1 indicating strong disagreement and 5 indicating strong agreement. In the present sample, the reliability of the BIS scale was McDonald’s ω = 0.70.

#### 2.2.2. Attentional Facilitation Index of Fear

The attentional facilitation index of fear was obtained using a dot-probe task. This procedure has been extensively used to assess attentional orientation toward emotions in both adults [25,26,57,58] and children [59]. In the dot-probe task, a series of picture pairs of a neutral face match with the same identity showing an emotional expression (e.g., a fearful face) on a black background, with one face on the left and the other on the right of a fixation cross separated by 6 cm. The duration of the face presentation was 500 ms, the faces were taken from the Dartmouth Database of Children’s Faces [60], and E-Prime 2.0 software [61] was used. After the faces pairs, a dot was displayed on the left or on the right of the fixation cross, and participants were requested to determine whether the dot appeared on the left or right, with the faces irrelevant to the task and to be ignored. Participants were requested to press a key on the keyboard corresponding to the location on the screen (left or right) where the dot-probe appeared as quickly as possible. If no key was pressed within 5000 milliseconds, the response was recorded as incorrect. Latency data for incorrect trials and when reaction times (RTs) were <100 ms or >5000 ms were not included in the calculation of facilitation indices. The task consisted of one block of practice stimuli (three neutral–neutral picture pairs) followed by six experimental blocks, each containing 40 face pairs (eight angry–neutral, eight fearful–neutral, eight happy–neutral, eight sad–neutral, and eight neutral–neutral) for a total of 240 face pairs. Angry, fearful, sad, happy, and neutral face pairs were pseudo-randomly presented within the blocks. A facilitation index (AFI) [62] was computed for each emotion type by subtracting the average RT to dot-probes replacing emotional faces in emotional neutral face pairs from the average RT to dot-probes replacing neutral faces in neutral–neutral face pairs: AFI = [(neutral-neutral-LEFT {probe LEFT} − emotional-neutral LEFT {probe LEFT}) + (neutral-neutral RIGHT {probe RIGHT} − emotional-neutral RIGHT {probe RIGHT})]/2. The measures of AFIs for each emotion were standardized. Positive AFI values indicate that facilitation (attentional capture) was due to the congruent emotional location, whereas negative AFI values would suggest the inhibition (avoidance) of congruent emotional locations compared to neutral baseline responses [63]. For the purpose of the present study, only the facilitation index for fearful faces was used.

#### 2.2.3. Awareness of Others’ Emotions

The awareness of others’ emotions was investigated using the scale of attending to others’ emotions of the self-reported Emotion Awareness Questionnaire (EAQ [64]; Italian version [65]); the scale was made up of 5 items (e.g., “It is important to know how my friends are feeling”) proposed on a 3-point Likert-type scale, from 1 (not true) to 3 (true). In the present sample, the reliability of this scale was McDonald’s ω = 0.74.

#### 2.2.4. Emotion Perception Accuracy of Fear

The Dartmouth Database of Children’s Faces [60], which is commonly used also to assess face and emotion recognition in children [66,67], was used in the present study. The models were photographed against a black background and wore black bibs and hats to conceal their hair and ears. The average estimated age of the chosen identities was 9.47 years (SD = 2.49). Each identity was represented by five emotional expressions, which were angry, fearful, sad, happy, and neutral. A total of 60 photos with 30° left-facing orientations were used in the present study. The faces were randomly presented on a black background for 500 ms within a rectangular frame measuring 8.5 cm by 5.5 cm. A 600 ms fixation cross intermixed the faces. Participants were instructed to identify the facial expressions by pressing one of five buttons on the keyboard. Prior to the main experiment, participants received training with three different faces from the Dartmouth Database of Children’s Faces, showing angry, neutral, and happy expressions. An index of emotion perception accuracy was computed following the method described by Fine and colleagues [68]; specifically, to compute the accuracy scores, the number of correct responses was squared, divided by the number of faces for each emotion, then multiplied by the number of times the participant identified each target emotion across all stimuli. These accuracy scores reflect how accurately participants identified each emotion set, adjusted for chance.

### 2.3. Data Analyses

First of all, descriptive statistics and zero-order correlations (i.e., Pearson’s *r*) for all study variables were calculated. To test the associations between the BIS and the Attentional facilitation index of fear, as well as the potential interactive role of Awareness of others’ emotions, Emotion perception accuracy of fear, and Gender, a hierarchical multiple regression approach was adopted. Specifically, Age (in months), Gender, BIS, Awareness of others’ emotions, and Emotion perception accuracy of fear were included in Step 1; two two-way interaction terms between the BIS and the other emotion-related variables (i.e., BIS × Awareness of others’ emotions and BIS × Emotion perception accuracy of fear) were added in Step 2; three two-way interaction terms with Gender (i.e., BIS × Gender, Awareness of others’ emotions × Gender, and Emotion perception accuracy of fear × Gender) were included in Step 3; and two three-way interaction terms (i.e., BIS × Awareness of others’ emotions × Gender and BIS × Emotion perception accuracy of fear × Gender) were tested in Step 4. For any significant interaction, the form of the interaction and significance of simple effects were explored using the post hoc probing procedures suggested by Holmbeck [69]. Specifically, as for continuous moderators, the regression equation derived from the full sample was used to estimate predicted values for the dependent variable at one SD below and one SD above the mean; as for gender, the regression was re-performed separating for boys and girls. Prior to computing interaction terms, scores of continuous variables were centered by subtracting the sample means.

## 3. Results

Descriptive statistics and zero-order correlations are reported in Table 1. At a zero-order level, the Attentional facilitation index of fear was unrelated to all other study variables.

Results of linear regression analyses are reported in Table 2. Once again, Step 1 revealed that the Attentional facilitation index of fear was unrelated to all other study variables. Nevertheless, two three-way interaction terms emerged in Step 4, indicating that—only in boys—the BIS was significantly and negatively associated with the Attentional facilitation index of fear at low (β = −0.40, *p* < 0.001) versus high (β = 0.06, *p* > 0.05) levels of Awareness of others’ emotions, as well as the BIS being significantly and negatively associated with the Attentional facilitation index of fear at low (β = −0.39, *p* < 0.01) versus high (β = 0.05, *p* > 0.05) levels of Emotion perception accuracy of fear.

## 4. Discussion

The extant literature has highlighted that youths with a high BIS (i.e., behavioral inhibition, an attentional system scanning for potential threats and regulating responses to negative stimuli) level are more likely to develop anxiety disorders [20,21,22,23], and some of them (~40%) go on to develop anxiety problems in later ages [23]. One possible processual mechanism calls into question the malfunctioning of the attentional facilitation to threats: while at normative levels, the facilitated detection of stimuli related to fear or threats is an adaptive biological response that may prepare the individual to quickly respond to threatening circumstances, at its high extreme, it becomes maladaptive and represents an attentional bias in which the individual promptly and preferentially attends to threatening stimuli compromising attentional performance [25,26,27,28]. Some studies have found that the increased detection of threat-related stimuli has been specifically linked to heightened anxiety, and it was proposed that attentional bias toward threats moderates the positive association between behavioral inhibition and anxiety-related outcomes [29,30,31,32]. Nevertheless, the association between behavioral inhibition and attentional bias to threats did not always emerge as significant [38,39,40].

The present study was specifically realized to further explore the association between youths’ level of behavioral inhibition and an attentional facilitation index of fear, considering the potential moderating role of two variables related to emotional competence (i.e., a self-reported awareness of others’ emotions and a measure of accuracy in recognizing fear arising from a laboratory task), as well as gender. The results revealed interesting associations that were not always in line with our hypotheses. First of all, both at the zero-order level and in the first step of our hierarchical regression (i.e., the main effects of predictors), the BIS was unrelated to the attentional facilitation index of fear. This is in line with those studies that found no associations between behavioral inhibition and the attentional bias to threat [38,39,40] and support the usefulness to test the presence of moderators.

**Figure 1:**
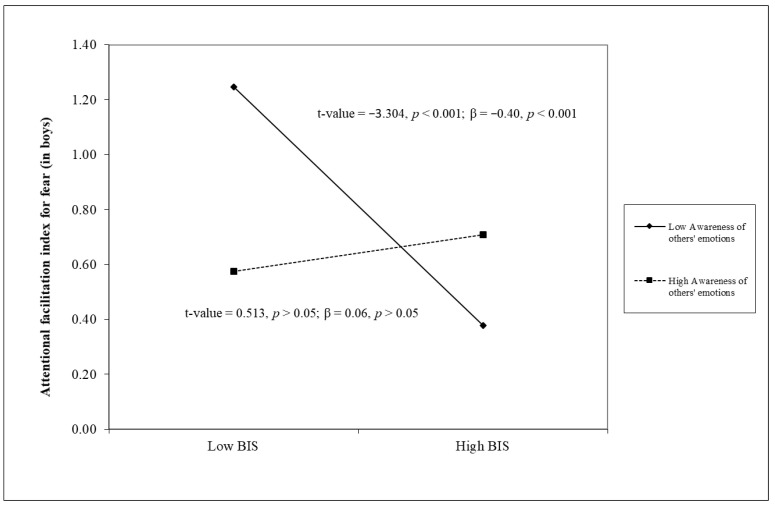
The moderating role of the awareness of others’ emotions in the association between the BIS and the attentional facilitation index for fear in boys.

**Figure 2:**
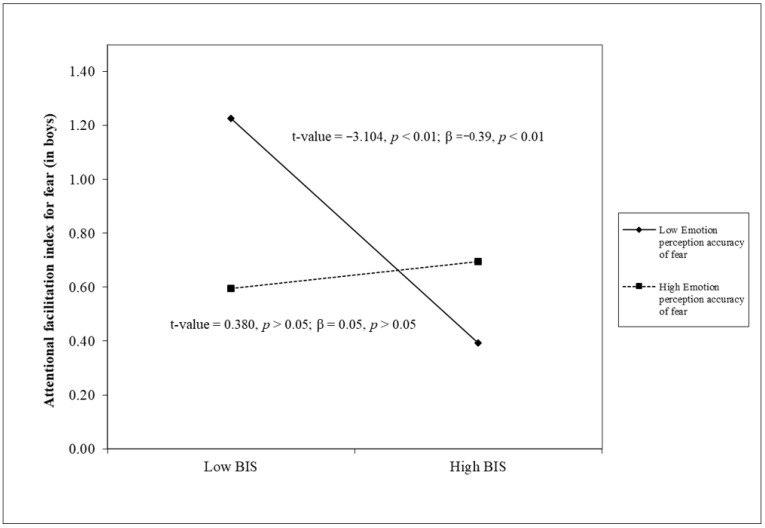
The moderating role of emotion perception accuracy of fear in the association between the BIS and the attentional facilitation index for fear in boys.

When we tested the potential moderating role of emotional competence and gender, two three-way interaction terms emerged. Specifically, only in boys, the BIS was significantly and negatively associated with the attentional facilitation index for fear at low versus high levels of self-reported awareness of others’ emotions or emotion perception accuracy of fear. As shown in Figure 1 and Figure 2, the level of the attentional facilitation index for fear was quite stable and at a medium level in those showing high emotional competence, irrespective of their level in the BIS; in contrast, at low levels of emotional competence, youths were more likely to present higher levels of attentional facilitation to fear only at low BIS scores. This pattern of findings would be consistent with the hypothesis that enhanced emotional competence could serve as a primary protective factor against attentional bias to threats (and probably against related anxiety, in line with the existing literature [70] by exerting a beneficial impact at both high and low levels of behavioral inhibition. Furthermore, our findings would indicate that the greatest risk for attentional bias to threats occurs in the compresence of low emotional competence and (contrary to our hypothesis) low behavioral inhibition; in other words, it would seem appropriate to strengthen youths’ emotional competence to avoid the development of biases toward threat, even in those who have low behavioral inhibition for whom part of the existing literature indicates a low risk of developing problems related to anxiety. Importantly, we obtain similar results considering in the same regression model a self-report measure and a laboratory task of emotional competence. This further corroborates the evidence that both the perception that individuals have of their own emotional functioning and their ability to actually recognize emotions (in the present study, fear) constitute protective elements that make a unique contribution to emotional development, which interventions or prevention programs must take into account. Finally, this study highlights that the above-discussed results emerged only in boys: as we can see in zero-order correlations reported in Table 1, girls showed higher levels of emotional competence, and this could have masked the protective role of this competence.

## 5. Limitations and Conclusions

All these results have to be read in light of some limitations. First, the cross-sectional nature of the present study did not allow us to establish causal conclusions, and future research should replicate these findings in a longitudinal study. Moreover, data were obtained within a community sample of middle school students; it should be interesting to test the same hypotheses within a clinical sample of youths who present anxiety disorders and maybe present higher levels of attentional facilitation to fear. Furthermore, our sample was quite homogenous with respect to ethnic backgrounds, as is typical in Italian schools; as a consequence, the generalizability of our results to other samples and countries should be tested. Finally, it should be important to replicate our results using not only self-report measures of the BIS but also with the concurrent use of behavioral indices in order to control social desirability.

In spite of the limitations, the results shed light on the moderating role of emotional competence and gender on the association between behavioral inhibition and attentional facilitation to fear. Consequently, this study stresses the importance of intervening in different aspects of emotional competence—both the perception that individuals have of their own emotional functioning and their actual ability to recognize emotions—in order to contribute to adaptive emotional functioning.

## Figures and Tables

**Table 1:** Descriptive statistics and zero-order correlations (Pearson’s *r*).

	M (SD)	Skew.	Kurt.	1	2	3	4	5	6
1—Age (a)	155.71 (10.69)	−0.26	−0.45	-					
2—Gender (b)	-	-	-	0.08	-				
3—BIS	3.29 (0.76)	−0.34	0.15	−0.08	−0.23 ***	-			
4—Attentional facilitation index of fear	0.00 (1.00)	−0.27	4.52	0.04	−0.02	−0.11	-		
5—Awareness of others’ emotions	2.55 (0.39)	−0.90	0.27	0.01	−0.30 ***	0.25 ***	−0.11	-	
6—Emotion perception accuracy of fear	0.50 (0.22)	−0.22	−0.59	0.09	−0.22 ***	0.08	−0.10	0.24 ***	-

Notes: *** *p* < 0.001. (a) Age is reported in months. (b) Kendall’s *tau-b* was used instead of Pearson’s *r* due to the dichotomous nature of gender.

**Table 2:** Linear regression analyses (standardized β).

	Age	Gender	BIS	Awareness of Others’ Emotions	Emotion Perception Accuracy of Fear	R^2^	F
Attentional facilitation index of fear	0.04	−0.09	−0.11 (a), (b)	−0.08	−0.10	0.01	(5263) = 1.686

Notes: Gender: 0 = girls, 1 = boys. (a) There is a three-way BIS × Awareness of others’ emotions × Gender interaction term: β = 0.27, *p* < 0.01; F (12,263) = 2.129, *p* < 0.05; R^2^ = 0.05, ΔR^2^ = 0.04, *p* < 0.01. The two-way BIS × Awareness of others’ emotions interaction term was significant in boys (β = 0.24, *p* < 0.01; F (6125) = 3.297, *p* < 0.01; R^2^ = 0.10, ΔR^2^ = 0.08, *p* < 0.01) but not in girls (β = −0.09, *p* > 0.05; F (6137) = 0.975; R^2^ = 0.001, ΔR^2^ = 0.001, *p* > 0.05). Specifically, the BIS was significantly and negatively associated with the Attentional facilitation index for fear at low (β = −0.40, *p* < 0.001) versus high (β = 0.06, *p* > 0.05) levels of Awareness of others’ emotions. (b) There is a three-way BIS × Emotion perception accuracy of fear × Gender interaction term: β = 0.21, *p* < 0.05; F (12,263) = 2.129, *p* < 0.05; R^2^ = 0.05, ΔR^2^ = 0.04, *p* < 0.01. The two-way BIS × Emotion perception accuracy of fear interaction term was significant in boys (β = 0.22, *p* < 0.05; F (6125) = 3.297, *p* < 0.01; R^2^ = 0.10, ΔR^2^ = 0.08, *p* < 0.01) but not in girls (β = −0.05, *p* > 0.05; F (6137) = 0.975; R^2^ = 0.001, ΔR^2^ = 0.001, *p* > 0.05). Specifically, the BIS was significantly and negatively associated with the Attentional facilitation index for fear at low (β = −0.39, *p* < 0.01) versus high (β = 0.05, *p* > 0.05) levels of emotion perception accuracy of fear.

## Data Availability

There are no unpublished data available. The corresponding author can be contacted regarding this matter.
